# Detection and characterization of bovine coronavirus and rotavirus in calves in Ethiopia

**DOI:** 10.1186/s12917-025-04563-9

**Published:** 2025-02-28

**Authors:** Yisehak Tsegaye Redda, Haileeyesus Adamu, Julia Bergholm, Johanna F. Lindahl, Anne-Lie Blomström, Mikael Berg, Tesfaye Sisay Tessema

**Affiliations:** 1 Department of Animal Biosciences, Swedish University of Agricultural Sciences, Box 7023, 750 07 Uppsala, Sweden; 2 Institute of Biotechnology, Addis Ababa University, P.O. Box 1176, Addis Ababa, Ethiopia; 3 Department of Animal Health and Antibiotic Strategies, Swedish Veterinary Agency, 751 89 Uppsala, Sweden; 4 College of Veterinary Sciences, Mekelle University, P.O. Box 231, Mekelle, Ethiopia

**Keywords:** Bovine coronavirus, Bovine rotavirus A, Calf diarrhea, Africa, qPCR, Genotyping

## Abstract

**Background:**

Bovine rotavirus A (BRVA) and bovine coronavirus (BCoV) cause significant diarrhea in young calves, leading to health issues and economic losses in the cattle industry. This study aimed to detect and molecularly characterize BRVA and BCoV in calves from Addis Ababa, Ethiopia. Fecal samples were collected from 105 calves under six months old, both with and without diarrhea. BRVA and BCoV were detected using quantitative real-time Polymerase Chain Reaction (qPCR), followed by genome sequencing for phylogenetic analysis and genotype determination.

**Results:**

BRVA was found in 3.8% of the calves, while BCoV was detected in 2.9%. The identified rotavirus genotypes included G10, found in diarrheic calves, and G8, found in a non-diarrheic calf. All BCoV infections occurred in diarrheic calves. Phylogenetic analysis of the BCoV spike protein 1 (S1) hypervariable region (HVR) and hemagglutinin esterase (HE) gene revealed close relationships with European and Asian strains. The S1 HVR of the current virus sequence PQ249423 was 100% identical at the nucleotide level to previously reported sequences from Ethiopia. Six amino acid substitutions in the HE gene of the current BCoVs were identified compared to the reference Mebus strain of BCoV. Phylogenetic analysis showed that the current G8 BRVA sequences clustered with bovine, caprine, and human rotavirus strains, while the G10 viruses formed a distinct cluster with bovine strains. The G10 viruses showed a 99.37% nucleotide sequence similarity to a previously reported BRVA from Ethiopia, and the G8 virus displayed the highest nucleotide similarity with a caprine isolate from India. Gene segment analysis of the current BRVA viruses indicated varying similarities with human, bovine, caprine, and porcine rotavirus strains, suggesting a potential reassortment event involving artiodactyl, human, and porcine rotavirus.

**Conclusions:**

This study demonstrates the presence of BRVA and BCoV in Ethiopian dairy calves and provides insights into their genetic diversity. Genetic analysis of BCoV revealed close relationships with strains from Europe and Asia. G10 and G8 were the identified BRVA genotypes, with G8 reported for the first time in Ethiopia. Future research should focus on broader sampling and molecular characterization to understand genetic diversity and devise effective control measures.

**Supplementary Information:**

The online version contains supplementary material available at 10.1186/s12917-025-04563-9.

## Introduction

Calf diarrhea is one of the most common and devastating diseases in the dairy industry worldwide. The multifactorial causes of calf diarrhea include infectious agents, nutritional deficiencies, environmental conditions, and management practices. Among the infectious agents, bovine group A rotavirus (BRVA) and bovine coronavirus (BCoV) are recognized as the most significant viral enteropathogens in acute diarrhea in young calves, leading to economic losses in the cattle industry worldwide [1, 2].

Rotaviruses, belonging to the *Reoviridae* family, are non-enveloped viruses with a genome of 11 segments of double-stranded RNA, enabling genetic reassortment and diversity among viral strains [3]. The 11 segments code for structural proteins (VP1, VP2, VP3, VP4, VP6, and VP7) and non-structural proteins (NSP1 to NSP6) [4]. Based on the genetic and antigenic properties of the VP6 protein, rotaviruses are classified into eight groups (A to H) [5]. Rotaviruses are further classified into genotypes based on genetic variations in the VP4 (P) and VP7 (G) genes [6]. Rotaviruses have been shown to infect a wide range of young species, including infants, various mammals (such as piglets, calves, goats, lambs, and foals), and birds [7]. BRVA is the most common viral pathogen in calf diarrhea [8, 9]. The virus primarily targets the small intestine, resulting in malabsorption and diarrhea, which leads to increased vulnerability to other infections, calf mortalities, and ultimately to economic losses due to slowed growth and treatment costs [10].

Another significant viral pathogen that can cause calf diarrhea is BCoV. It has a single-stranded positive-sense RNA (ssRNA) genome and shows considerable genetic diversity. BCoV's genetic diversity is likely driven by a combination of factors, including frequent replication cycles, immune selection pressure, and recombination events, in addition to mutations [11–14].

BCoV affects both young and adult cattle, causing acute diarrhea in calves and winter dysentery in adults, resulting in substantial economic losses in dairy and beef herds worldwide [15, 16]. BCoV falls into the beta group of coronaviruses, including viruses such as SARS-CoV, MERS-CoV, HCoV-OC43, and SARS-CoV-2. These viruses are significant human pathogens with zoonotic origins and the latter two are globally prevalent [17, 18]. Additionally, bovine-like coronaviruses have been implicated in enteric and respiratory diseases across various ruminant species, dogs, and humans, indicating potential cross-species transmission [19].

The dairy farming sector in Ethiopia is crucial to the country's agricultural economy, supporting the livelihoods of many smallholder farmers [20]. Calf diarrhea is a major health concern in the Ethiopian dairy industry, leading to significant economic losses and being the primary cause of neonatal mortality in calves, accounting for 18% to 63% of deaths [21, 22]. While BRVA and BCoV are known to play a role in calf diarrhea, there has been limited research on their prevalence and genetic diversity in Ethiopia. Understanding the epidemiology and genetic characteristics of these viruses is crucial for developing effective disease control strategies. Therefore, the purpose of this study was to genetically characterize BRVA and BCoV circulating in calves in Addis Ababa, Ethiopia.

## Results

### Detection and characterization of BCoV

Among the 105 samples tested using BCoV qPCR, 2.9% (3/105) tested positive. All positive samples came from diarrheic calves, with 7.1% (3/42) of the diarrheic calves testing positive. Two of the positive samples, ETB_14 and ETB_47 were obtained from 1 and 2-month-old calves residing on the same property in Bole sub-city. The third positive sample, ETB_94, was from a 1-month-old calf on a farm located in the Yeka Sub-city.

Sample with cycle threshold (Ct) values less than 40 were considered positive. The Ct values for the current positive samples were 31.79 for ETB_14, 32.17 for ETB_47, and 37.10 for ETB_94. Of the three samples, only one (ETB_14 with a Ct value of 31.79) was successfully amplified by the nested PCR targeting the S1 HVR.

**Fig. 1:**
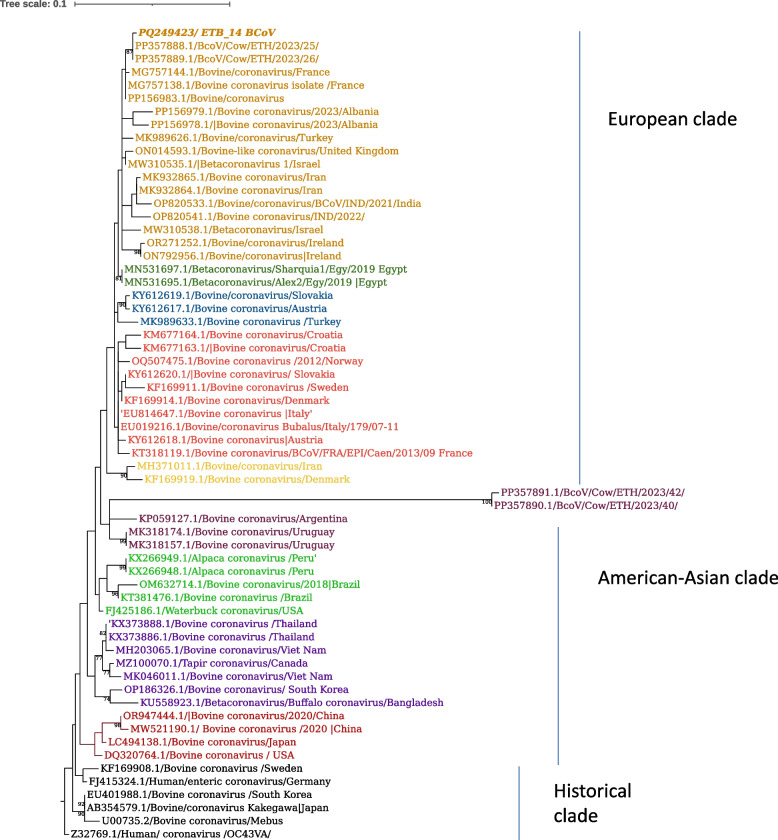
Maximum likelihood phylogenetic tree of S1 HVR sequences of BCoV. The sequences of the present study are in bold and italicized. Each color represents a specific clade. The human enteric coronavirus strain OC43VA served as the outgroup. The scale bar at the top of the tree calibrates the genetic distance expressed as nucleotide substitution per site. Bootstrap values > 70% are displayed

To investigate the relationship between the viruses detected to other BCoV, the PCR product of S1 HVR was sequenced. The 451 bp S1 HVR sequence (PQ249423) from our study showed 99.8–100% nucleotide sequence similarity with the previously reported sequences from Ethiopia (PP357889.1 and PP357888.1). Furthermore, the phylogenetic analysis showed that the current virus PQ249423 clusters together with strains from Ethiopia (PP357889.1 and PP357888.1) as well as from other European and Asian countries (Fig. 1). On the other hand, two other viral sequences previously reported from Ethiopia (PP357891.1 and PP357890.1) and the current virus PQ249423 are grouped in different clusters (Fig. 1). A 94.49% nucleotide identity to the Mebus BCoV strain, a widely used reference strain known for its well-characterized genome and biological properties, and a 95.36% nucleotide similarity to the OK-0514–3 strain, a human enteric coronavirus, were observed.

**Fig. 2:**
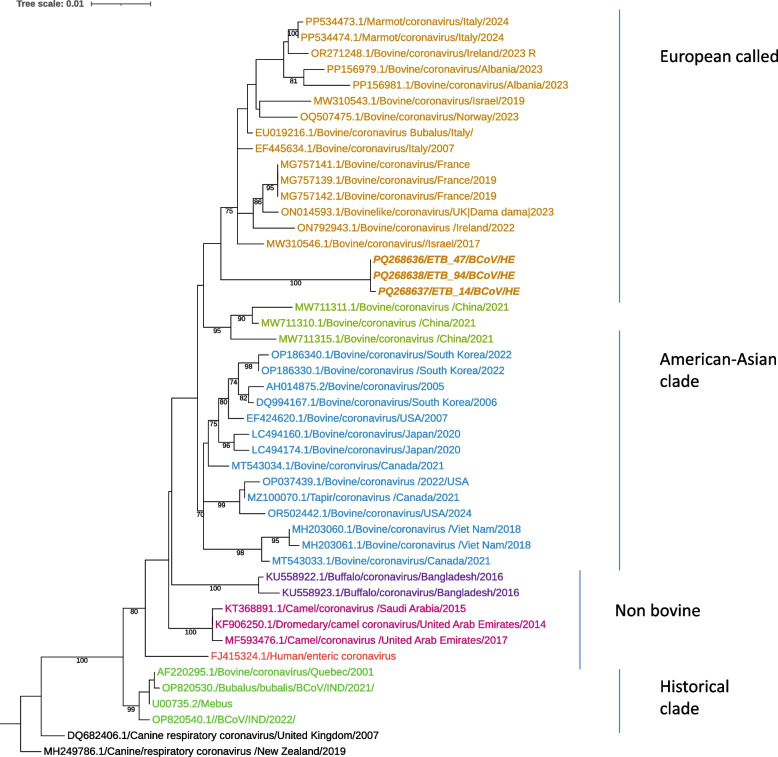
Maximum likelihood phylogenetic tree of HE sequences of bovine coronavirus. The strains of the present study are bold and italicized. Each color represents a specific clade. The canine respiratory coronavirus MH249786 served as the outgroup. The scale bar at the top of the tree calibrates the genetic distance expressed as nucleotide substitution per site. Bootstrap values > 70% are displayed

We next analyzed the hemagglutinin-esterase (HE) gene. The HE gene from all three positive samples were successfully amplified and sequenced. A phylogenetic tree constructed using the 1216 bp partial sequence of HE genes demonstrated that the three sequences from this study, ETB_47/BCoV/HE (PQ268636), ETB_14/BCoV/HE (PQ268637), and ETB_94 BCoV/HE (PQ268638), clustered together with other BCoV HE genes from Europe and the Middle East in one clade (Fig. 2). The viral sequences from the current study formed a distinct group within the clade. Comparison of the HE genes among the three currently identified sequences revealed a high degree of similarity, with 99.9%–100% nucleotide identities. BLASTn analysis of the HE gene showed that the three viruses had the highest nucleotide sequence identities (97.73%) with a BCoV strain from France (MG757141). Additionally, the HE gene of current BCoVs exhibited 96.8% nucleotide identity with the reference strain Mebus (U00735.2).

Amino acid analysis of the HE genes of the three viruses identified in the present study, compared to the reference Mebus strain, revealed six sites of amino acid substitutions (S156A, L309P, N357D, P367S, N379T, S380P) (Table 1).

**Table 1:** Variations in the HE glycoprotein amino acid sequences of BCoV-positive samples compared to the reference Mebus strain^a^

BCoV / amino acid position	156	309	357	367	379	380
PQ268638 (ETB_94 BCoV/HE)	S	L	N	P	N	S
PQ268636 (ETB_47 BCoV/HE)	S	L	N	P	N	S
PQ268637 (ETB_14 BCoV/HE)	S	L	N	P	N	S
**AAA66393.1_ Mebus**	**A**	**P**	**D**	**S**	**T**	**P**

^a^The letters represent different amino acids

### Detection and characterization of BRVA

Out of 105 samples, 3.8% (4/105) tested positive for BRVA by using a qPCR approach. Samples with Ct values less than 36 were considered positive. The current samples had Ct values ranging between 27.75–33.2. Two rotaviruses (ETB_02 and ETB_66) were of the G10 genotype, originating from diarrheic calves. Of the two remaining qPCR-positive samples, one (ETB_53) had the G8 genotype, originating from a non-diarrheic calf, while the rotavirus ETB_89 remained untyped.

**Fig. 3:**
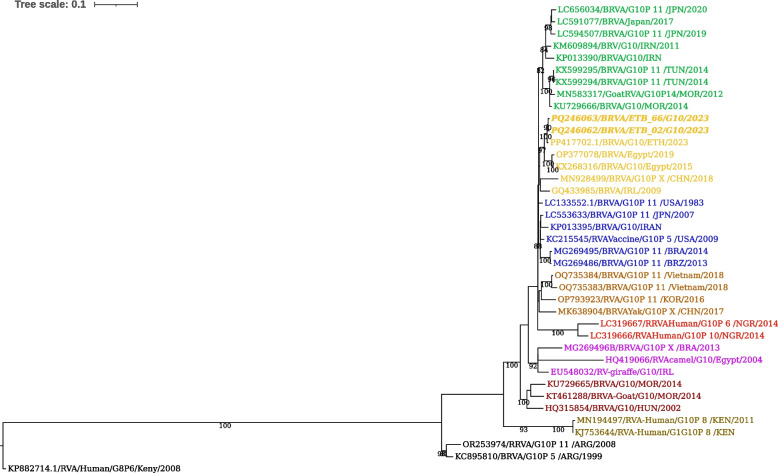
Maximum likelihood phylogenetic tree of G10 sequences of BRVA. The G8 human RVA strain KP882714.1 served as the outgroup. The strains of the present study are indicated in bold and italicized. Each color represents a specific clade. The scale bar at the top of the tree calibrates the genetic distance expressed as nucleotide substitution per site. Bootstrap values > 70% are displayed

Phylogenetic analysis of the 821 bp partial sequence of the G10 genotypes was conducted, including the G10 sequences of ETB_02 (PQ246062) and ETB_66 (PQ246063) detected in this study. ETB_02 and ETB_66 formed a monophyletic group together with BRVA G10 from Ethiopia (PP417702.1), Egypt (KX268316 and OP377078), China (MN928499), and Ireland (GQ433985) (Fig. 3). The current two viruses were 99.5% identical to each other at the nucleotide level. Additionally, the current G10 sequences were closely related to bovine rotavirus previously reported from Ethiopia (PP417702.1), sharing 99.37% nucleotide identity.

**Fig. 4:**
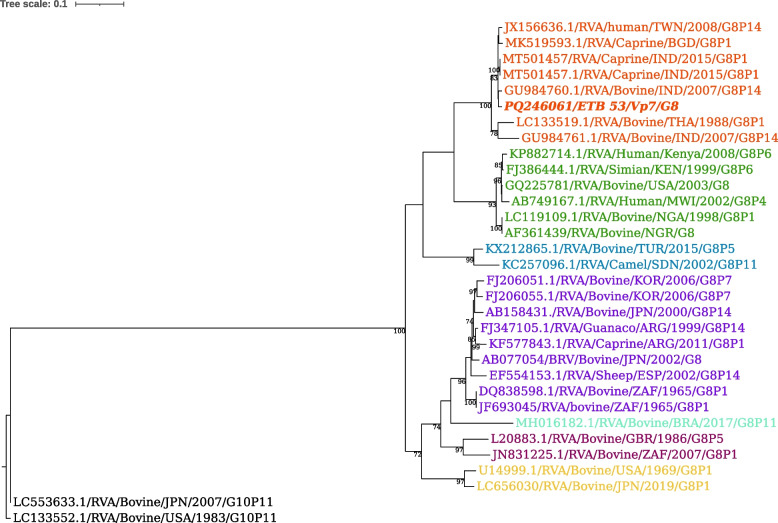
Maximum likelihood phylogenetic tree of G8 sequences of bovine rotavirus A. The strains of the present study are indicated in bold and italicized and each color represents a specific clade. The G10 bovine RVA strain LC133552.1 served as the outgroup. The scale bar at the top of the tree calibrates the genetic distance expressed as nucleotide substitution per site. Bootstrap values > 70% are displayed

Phylogenetic analysis of the 830 bp partial nucleotide sequences of representative G8 Bovine rotavirus showed that the current virus ETB_53 (PQ246061) clustered with bovine rotavirus G8 from India (GU984760.1), Thailand (LC133519.1), Caprine rotavirus from Bangladesh (MK519593.1) and India (MT501457), and human rotavirus from Taiwan (JX156636.1) (Fig. 4). ETB_53 showed the highest nucleotide sequence similarity of 98.68% with a caprine rotavirus from India (MT501457.1).

Seven of the 11 rotavirus genes, including VP1, VP2, VP6, VP7, NSP2, NSP4, and NSP5, were successfully sequenced from the three positive samples, while the VP4, NSP1, NSP3, and VP3 genes could not be amplified. The nucleotide sequence analysis in ViPR and BLASTn analysis of each gene revealed the following genotype constellations: G8P[X]-I2-R2-C2-Mx-Ax-N2-Tx-E2-H3 for the ETB_53 BRVA, and G10P[X]-I2-R2-C2-Mx-Ax-N2-Tx-E2-H3 for the ETB_02 and ETB_66 BRVA.

The ETB_53 BRVA segments VP1, VP2, VP4, VP6, NSP2, and NSP4 had the closest nucleotide sequence identity with human rotavirus strains from different parts of the world (Table 2). Interestingly, the NSP2 had the closest nucleotide sequence identity with a previously reported human rotavirus from Ethiopia (KP752895). The NSP5 had the closest nucleotide sequence identity with OR270741, isolated from a chamois in Slovenia. For ETB_66 and ETB_02, the VP1 and VP2 sequences had the closest nucleotide sequence identity with human rotavirus, VP6 and NSP5 had the closest nucleotide identity with bovine isolates, and lastly, the NSP2 and NSP4 had the closest nucleotide identity with porcine rotavirus strains (Table 2).

**Table 2:** Genotyping based on seven gene segments and the closest nucleotide sequence's accession number for the current BRVA gene segments

	**ETBRV_53**		**ETBRV_66**		**ETBRV_02**	
Segment	Genotype	Similarity^a^	Genotype	Similarity^a^	Genotype	Similarity^a^
VP7	G8	MT501457.1 98.68% Caprine India	G10	PP417702.1 99.37% Bovine Ethiopia	G10	PP417702.1 99.37% Bovine Ethiopia
VP1	R2	ON737422.1 98.58% Human USA	R2	MG214337.1 99% Human Morocco	R2	MG214337.1 92% Human Morocco
VP2	C2	KJ187600 95% Human South Korea	C2	GU296422.1 95% Human Italy	C2	KU317467.1 95.45% Human Egypt
VP6	I2	KJ752132.1 98% Human Ethiopia	I2	OL988984.1 97% Bovine Ireland	I2	OL988984.1 100% Bovine Ireland
NSP2	N2	KP752895.1 97% Human Ethiopia	N2	AB972863.1 98% Porcine Thailand	N2	AB972863.1 98% Porcine Thailand
NSP4	E2	FN665697.1 95% Human Hungary	E2	AB972865.1 97.7% Porcine Thailand	E2	AB972865.1 97.63% Porcine Thailand
NSP5	H3	OR270741.1 96.16% Chamois Slovenia	H3	JX402800.1 98.62% Bovine Slovenia	H3	JX402800.1 98.62% Bovine Slovenia

^a^The closest nucleotide sequence accession number, sequence identity (in %), host organism ( species), and sequence origin (Country)

## Discussion

The present study aimed to analyze the molecular diversity of bovine coronavirus (BCoV) and bovine group A rotavirus (BRVA) identified from calves in Addis Ababa, Ethiopia. The study found that the overall detection rate of BCoV in calves was 2.9%; all positive samples originated from diarrheic calves (7.1%). This suggests that BCoV might not be widespread in the general calf population but is more associated with diarrhea. Other studies have reported BCoV prevalence in diarrheal feces ranging from 3.4% to 69% [23]. Various studies also indicated a higher detection rate of BCoV in diarrheic calves compared to healthy ones [24–27]. This supports a strong link between BCoV infection and calf diarrhea.

Additionally, the Ct values for the current samples ranged from 31.79 to 37.10, indicating a low viral load. This suggests that BCoV can cause diarrhea even when viral particle concentrations in the gut are low. However, it is important to note that BCoV was only detected in 7.1% of the diarrhea cases examined. This low detection rate implies that other pathogens or factors could also be responsible for causing diarrhea in the majority of cases.

The consistently low detection rates of BCoV in Ethiopian calves, ranging from 0.91% to 8.5% in this and previous studies [28–30], may be influenced by environmental, management, breed, and geographical factors. Larger studies and detailed epidemiological analyses are necessary to better understand its prevalence and impact.

The hemagglutinin-esterase (HE) and Spike (S) proteins are crucial attachment proteins of BCoV, essential for the virus invasion and release from host cells, serving as critical antibody-neutralization epitopes [31] The S1 hypervariable region (HVR) is particularly significant for virus-host interactions, providing valuable insights into the genetic diversity and evolutionary relationships of BCoV strains. Additionally, the HE gene plays an important role in viral entry and spread [32–34] and its genetic characterization is crucial for enhancing our understanding of BCoV evolution. Recent studies have underscored its importance in identifying novel emerging strains [35].

The S1 HVR gene sequence of ETB_14 (PQ249423), showing 99.8–100% nucleotide sequence similarity with previously reported sequences from Ethiopia (PP357889.1 and PP357888.1), indicates a high level of genetic conservation among these strains.

The phylogenetic analysis of the S1 HVR showed that the current virus sequence PQ249423, clustered with strains from diverse geographical regions, including the Middle East, Asia, and Europe (Fig. 1). This clustering indicates that BCoV strains circulating in different parts of the world share significant genetic similarities, possibly due to regional transmission or common ancestry. It could also be due to the limited inherent mutations within the virus, as coronaviruses have a relatively moderate mutation rate due to their proofreading mechanism [14]. The current virus sequence exhibited 94.49% nucleotide identity with the reference Mebus strain and 95.36% similarity to the human enteric coronavirus strain OK-0514–3, highlighting its relatively limited divergence from these reference strains. The reference strain Mebus occupied a separate branch, supporting the observed genetic distance and divergence. This data is consistent with several reports describing the genetic distance between the reference Mebus strain, a widely used and well-characterized reference strain isolated in the 1970s from a calf with neonatal diarrhea, and newer BCoV strains [36–38]. On the other hand, two previously reported BCoVs from Ethiopia (PP357891 and PP357890) and the current BCoV are grouped in different clusters (Fig. 1), indicating that at least two different stains are circulating in the country [29].

Phylogenetic analysis of the HE genes revealed that the current sequences PQ268636, PQ268637, and PQ268638 clustered together with other BCoV HE genes from Europe and the Middle East (Fig. 2). This suggests a close genetic relationship between the current viruses and the European and Middle East strains indicating possible regional transmission or common ancestry. The HE gene of the current viruses exhibited a high nucleotide sequence identity ranging from 99.9%-100% among themselves, suggesting a recent common origin and the circulation of closely related genetic variants in the study population.

The analysis of amino acids in the HE genes revealed six substitution sites (S156A, L309P, N357D, P367S, N379T, S380P) compared to the Mebus strain. These substitutions may be linked to virulence, as all current sequences were detected from diarrheic cases. This can be supported by the findings of Zhang et al. [39], who reported amino acid substitutions of HE proteins between the avirulent BCV-L9 and virulent BCV-LY138 at positions P367S and N379T. David et al. [35] also documented differences in L309P and S380P between winter dysentery and the Mebus strain. Furthermore, variations in the HE gene were clustered in the major regions (amino acids 309–380), which included the membrane-proximal domain and the putative esterase domain of the HE gene [40]. This suggests that these regions are subjected to selective pressure and might affect viral–host interactions. The current HE sequences from this study do not exhibit frameshifts, deletions, or insertions, unlike the novel emerging strains reported from China and the United States. Lang et al. [31] indicated that HE and S are integral components, and when HE undergoes a critical mutation, it induces S mutations and strengthens its affinity.

The detection of BRVA in 3.8% of the samples in this study indicates that the virus is present in the cattle population within the studied area, though at a relatively low level. The presence of rotavirus in both diarrheic and non-diarrheic calves suggests that the virus may exhibit varying degrees of pathogenicity [41]. Additionally, it implies that factors such as the immune status of the calves or environmental conditions may influence whether the virus results in diarrhea [42, 43]. The sample from non-diarrheic calves contains a low viral load with a Ct value of > 32.5, indicating that the virus is present in such small quantities that it may not be sufficient to cause noticeable illness.

The detection of multiple BRVA genotypes (specifically G10 and G8) within the population provides valuable insights into the diversity of BRVA strains in the study area. To our knowledge, this is the first G8 BRVAs genotype report from Ethiopia. Understanding the prevalence of different genotypes is crucial because they can exhibit varying levels of virulence, influence immune responses, and impact vaccine efficacy. The G8 and G10 genotypes are common rotavirus A strains of bovine origin and have been linked to interspecies transmission between cattle and humans.

Phylogenetic analysis of the G10 strains (Fig. 3) showed that the current viruses sequence PQ246062 and PQ246063 clustered together with other G10 BRVA from diverse geographical regions, including Egypt, China, Ireland, and the USA, emphasizing their genetic similarity and potential evolutionary connections. The current G10 genotype viruses exhibited a 99.5% nucleotide similarity with each other and a 99.37% similarity with previously reported sequences from Ethiopia. This high genetic similarity suggests potential epidemiological connections or a shared ancestry among the current G10 genotype circulating in the country.

The phylogenetic analysis of nucleotide sequences of representative G8 genotypes (Fig. 4) revealed that the current BRVA, ETB_53 (PQ246061), clustered with bovine, caprine, and human rotavirus strains from various Asian countries (India, Thailand, Bangladesh, and Taiwan). This clustering suggests the possibility of cross-species transmission or a shared evolutionary origin. Studies have shown that G8 rotaviruses have been isolated from various hosts, including cattle, caprine, and even humans, indicating cross-species transmission [44–47].

A nomenclature for the comparison of complete rotavirus genomes was considered in which the notations Gx-P[x]-Ix-Rx-Cx-Mx-Ax-Nx-Tx-Ex-Hx are used for the VP7-VP4-VP6-VP1-VP2-VP3-NSP1-NSP2-NSP3-NSP4-NSP5/6 encoding genes, respectively . In the current study, seven out of the 11 rotavirus genes, VP1, VP2, VP6, VP7, NSP2, NSP4, and NSP5 were successfully sequenced from three positive samples, while VP4, NSP1, NSP3, and VP3 could not be amplified. This could be attributed to low-quality samples, mutations in the primer binding regions, or the high genetic diversity of VP4 and NSP1 compared to more conserved genes such as VP6 and NSP2 [48].

The genotype constellation based on the seven genes of the current BRVA strains ETB_66 and ETB_02 was G10P[X]-I2-R2-C2-MX-AX-N2-TX-E2-H3. Similarly, ETB_53 exhibited G8P[X]-I2-R2-C2-MX-AX-N2-TX-E2-H3. This pattern is observed in both human and artiodactyl (bovine, caprine, and ovine) species of rotaviruses [47, 48], suggesting potential cross-species transmission.

Genomic segments in ETB_53 had the closest nucleotide similarity to human rotavirus isolates from various geographic regions (Table 1). Notably, the NSP2 segment of ETB_53 was most closely related to human rotavirus from Ethiopia (KP752895); this suggests a potential genetic link or shared evolutionary pathway between bovine and human rotaviruses, possibly due to interspecies transmission events or common viral ancestors. Evidence of naturally occurring reassortment events between RVA strains of animal and human origin has been demonstrated by various researchers [47–53]. Additionally, the NSP5 and VP7 segments of ETB_53 exhibited the highest nucleotide similarity with an isolate from a chamois in Slovenia and a caprine from India, respectively. This finding suggests that these viruses may have originated from reassortment events involving rotaviruses from artiodactyls and humans.

Similarly, the genetic segment analysis of the G10 viruses (ETB_66 and ETB_02) highlights intricate relationships among rotaviruses from various host species, including bovine, human, and porcine (Table 2). The mixed genetic structure of these viruses suggests that they may have originated from multiple reassortment events involving rotaviruses from different hosts, such as humans, cattle, and pigs. The proximity or shared living spaces between animals and humans facilitates the transmission of rotaviruses across species, especially in areas with low socioeconomic conditions [54]. A study conducted in Uganda [55] found that complex genome reassortment events occur between rotavirus strains from humans, cattle, goats, and pigs. This suggests that reassortment is a common mechanism influencing the evolution and epidemiology of these viruses.

This study has certain limitations, including a small sample size, restricted geographic scope, and short collection period. These factors limit its ability to fully represent the broader population or capture seasonal and temporal trends in BCoV and BRVA circulation, potentially affecting the generalizability of its findings. Despite these limitations, the study provides valuable insights into the molecular characterization of BCoV and BRVA in Ethiopian calves, offering critical data on circulating virus strains where such information is scarce. Future studies should address these gaps by using larger, more diverse samples and extending study durations.

## Conclusions

In conclusion, this study highlights the detection of both BRVA and BCoV in dairy farms around Addis Ababa, Ethiopia. While the prevalence of these main calf enteric pathogens was not very high in the study area, molecular analysis revealed notable findings. The current BCoV exhibited close genetic relationships with strains from Europe and other regions, with notable amino acid substitutions in the HE genes that could be linked to increased virulence. Furthermore, G10 and G8 were identified as BRVA genotypes, with G8 being reported for the first time in Ethiopia. Genetic analysis suggests the current BRVA strains likely arose from multiple reassortment events involving rotavirus from different host species. The findings underscore the importance of genetic characterization for detecting emerging strains that could impact animal health or have zoonotic potential. The presence of BCoV strains with amino acid substitutions and the emergence of the G8 BRVA genotype in Ethiopia highlights the need for continued surveillance and monitoring of these pathogens. Future research should focus on broader sampling and molecular characterization to better understand the genetic diversity of BCoV and BRVA, which will be crucial for devising effective control measures.

## Methods

### Study design and sample collection

**Fig. 5:**
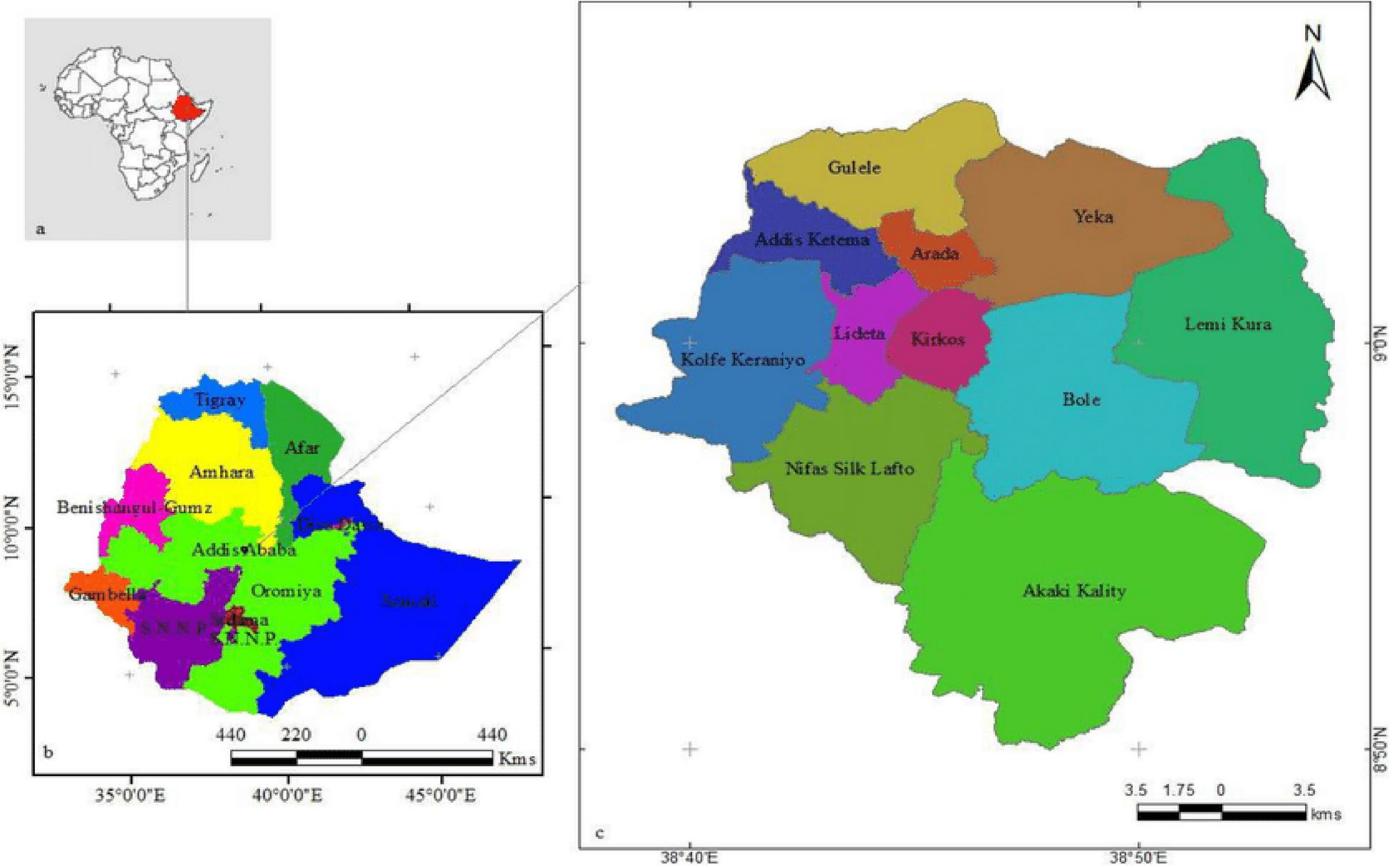
Map of Addis Ababa with Sub Cities

A cross-sectional study was conducted between October to December 2023. A total of 105 fecal samples, 42 from diarrheic and 63 from apparently healthy animals, were collected from crossbreed (Zebu x Holstein–Friesian) calves (less than 6 months old) from 67 smallholder dairy farms located in 9 sub-city in Addis Ababa (Gulele, Yeka, Lemi kura Akaki Kality, Nifasilk Lafto Kolfe Keraniyo, Bole and Kirkos) (Fig. 5). These smallholder dairy farms are managed by individuals, families, and small and micro-enterprises (SMEs), with herd sizes in these farms was range from 1 to 27 cattle. Consent of the owners was obtained to collect fecal samples from their calves.

The diagnosis of diarrhea and sample collection was made by a veterinarian during the survey. Diarrhea was considered if the feces were loose to watery, with or without the presence of blood and mucus. Fecal samples were collected directly from the rectum in sterile stool cups and transported under a cold chain from the field to the Institute of Biotechnology, Addis Ababa University (AAU), where the samples were stored at -20°C until further processing.

### Nucleic acid extraction

Each sample was diluted 1:10 in phosphate-buffered saline (PBS) and then centrifuged at 10,000 rpm for 10 min. The supernatant was collected and stored at -80 °C for future analysis. Total RNA was extracted as described by [56]. Briefly, 250 µl of supernatant was mixed with 750 µl of TRIzol reagent. After a 5-min incubation at room temperature, 150 µl of chloroform was added, followed by vigorous vortexing for 15 s. The tubes were centrifuged at 12,000 rpm for 15 min at 4 °C. The upper aqueous phase was transferred to a 1.5 ml tube. An equal volume of 70% ethanol was added, and the remaining RNA extraction was conducted using GeneJET RNA purification kit (Thermo Fisher Scientific), according to the manufacturer’s instructions. The extracted RNA was stored at -80 °C until further use.

### qPCR based detection of bovine coronavirus (BCoV)

cDNA was synthesized using the SuperScript IV cDNA synthesis kit (Thermo Fisher Scientific) and random hexamers following the manufacturer’s instructions. The samples were screened for BCoV using qPCR primers targeting the M-gene of bovine coronavirus, as described by [57]. Briefly, PCR reactions were set up in a 20 μl volume containing 1X iTaq™ Universal Probes Supermix (2x) (Bio-Rad), 600 nM of each primer (BCoV-F and BCoV-R), 200 nM of probe (BCoV-Pb) (Table 3), and 2 µl of cDNA template. The thermal cycling program consisted of 95 °C for 5 min initial denaturation, followed by 95 °C for 15 s and 60 for 1 min repeated for 45 cycles*.* The qPCR was performed on the CFX96 Real-Time PCR System (Bio-Rad); a cutoff (Ct)value < 40 was considered positive.

**Table 3:** List of primers and probes

gene	Primer	Primer/probes sequence (5’-3’)	Product size-bp	Reference
VP6	VP6-F	GGCTTTWAAACGAAGTCTTC	928	[58]
	VP6-R	GGYGTCATATTYGGTGG		
VP7	VP7-F	ATGTATGGTATTGAATATACCAC	881	[59]
	VP7-R	AACTTGCCACCATTTTTTCC		
NSP2	NSP2-F	GGCTTTTAAAGCGTCTCAGTC	1059	[60]
	NSP2-R	GGTCACATAAGCGCTTTCTAT		
NSP4	NSP4-F	GGCTTTTAAAAGTTCTGTT	750	[60]
	NSP4-R	GGTCACRYTAAGACCRTTCC		
NSP5	NSP5-F	GGCTTTTAAAGCGCTACAGTG	664	[61]
	NSP5-R	GGTCACAAAACGGGAGTG		
VP1	VP1-F	GGCTATTAAAGCTGTACAATGGG	686	[52]
	VP1-R	TAATCCTCATGAGAAAACATGAC		
VP2	VP2-R	CTTCATCTTGAAATATAGCATCAC	686	[52]
	VP2-F	GGCTATTAAAGGCTCAATGGCG		
HE	HEA-F	CAGTGAAGAAGACTAAACTCAGT	741	[53]
	HEA-R	TAAATAACACCAGTGTCTTTATT		
	HEB-F	TGACGAGTATATCGTACCACTT	684	
	HEB-R	CTAAGCATCATGCAGCCTAGTACC		
S1 HVR	S1HS-F	CTATACCCAATGGTAGGA	885	[62]
	S1HA-R	CTGAAACACGACCGCTAT		
	S1NS-F	GTTTCTGTTAGCAGGTTTAA	488	
	S1NA-R	ATATTACACCTATCCCCTTG		
BCoV M gene	BCoV-F	CTGGAAGTTGGTGGAGTT	85	[57]
	BCoV-R	ATTATCGGCCTAACATACATC		
	BCoV-Pb	FAMd -CCTTCATATCTATACACATCAAGTTGTT-BHQ1		
BRVA NSP5	Rota-F	TGATTCTGCTTCAAACGATCCA	65	[29]
	Rota-R	GCATTTGTCTTAACTGCATTCGA		
	Rota-Pb	VIC-TCACCAGCTTTTCGATAAG-MGB		

### qPCR detection of bovine rotavirus (BRV)

The samples were screened for rotavirus using a qPCR targeting the NSP5 gene of BRV as described by [29]. A 30 µl reaction volume was prepared, consisting of 1X Taqman Fast Virus 1-Step Master Mix (Applied Biosystems), 600 nM of forward and reverse primers (Table 3), 150 nM of probe, and 2 µl of sample RNA. The PCR program used was as follows: 50 °C for 5 min, 95 °C for 20 s, then 45 cycles of 95 °C for 15 s, 60 °C for 1 min, followed by a plate read. The qPCR was performed on the CFX96 Real-Time PCR System (Bio-Rad); cutoff (Ct) Value < 36 was considered as positive.

### Genetic characterization of BCoV and BRVA

PCR amplification of the target BRVA (VP7, VP6, VP1, VP2, NSP2, NSP4, and NSP5) genes was performed using various primer sets (Table 3) and the 2X Platinum SuperFi PCR Master Mix (Invitrogen). Each 20 µL reaction mixture contained 1X of the 2X Platinum SuperFi PCR Master Mix (Invitrogen), 600 nM of both forward and reverse primers, and 2 µL of cDNA. The PCR cycling conditions were as follows: initial denaturation at 98 °C for 30 s, followed by 35 cycles of 98 °C for 10 s, 60 °C for 30 s, and 72 °C for 30 s, with a final extension at 72 °C for 5 min. The PCR products were then analyzed on 1–2% agarose gels stained with GelRed™ and visualized using the ChemiDoc™ MP Imaging System (Bio-Rad®). Similarly, the S1 hypervariable region (S1 HVR) of BCoV was amplified using nested PCR-specific primers described by [62], and the HE gene was amplified using overlapping primers as described by [30] using the above procedure.

The PCR products were purified using the GeneJET Gel Extraction Kit (Thermo Fisher Scientific) according to the manufacturer’s instructions. After purification, the samples were sent to Macrogen Europe for Sanger sequencing. The quality of the sequences was analyzed, and the consensus sequences were assembled using Geneious Prime (v.2024.0.7). The sequences were then submitted to the Basic Local Alignment Search Tool (BLAST) (http://blast.ncbi.nlm.nih.gov/Blast.cgi) to identify homologous strains. In addition, the genotypes of the genome segments of the rotavirus were identified using the viral species identification tool available in the Virus Pathogen Database and Analysis Resource (ViPR) [63].

### Phylogenetic analysis

The nucleotide sequences of BRVA and BCoV were aligned with representative sequences from GenBank using the ClustalW algorithm in MEGA X [64]. Phylogenetic trees were constructed using MEGA X software [64]. For each sequence dataset, the best model was determined using the “Find Best DNA/Protein model” application, and the trees were built using the suggested models, with 1,000 bootstrap replicates, and a support threshold of 70%. The phylogenetic trees were based on the Maximum Likelihood (ML) method, with the following specific models assigned to the different datasets: TN93 + G for BCoV S1 HVR gene, GTR + G + I for BCoV HE gene, T92 + G for the rotavirus G10 VP7 gene, and GTR + G for G8.

Additionally, the deduced amino acid sequences of the HE gene of the current BCoVs were aligned with the BCoV Mebus strain using the Jalview alignment tool (v2.11.3.3), and the amino acid differences were identified.

## Supplementary Information

Supplementary Material 1.

## Data Availability

The nucleotide sequences of the gene segments of the current isolates are available at NCBI GeneBank under accession no. PQ246055-PQ246066, PQ279786-PQ279788(BRVA) and PQ268636-PQ268638, PQ249423(BCoV). The datasets used and analyzed during the current study are available from the corresponding author on reasonable request.

## References

[1] Barry AF, Alfieri AF, Stipp DT, Alfieri AA. Bovine coronavirus detection in a collection of diarrheic stool samples positive for group a bovine rotavirus. Braz Arch Biol Technol. 2009;52(spe):45–9.

[2] Gulliksen SM, Jor E, Lie KI, Hamnes IS, Løken T, Åkerstedt J, et al. Enteropathogens and risk factors for diarrhea in Norwegian dairy calves. J Dairy Sci. 2009;92(10):5057–66. 19762824 10.3168/jds.2009-2080 PMC7094401

[3] McDonald SM, Nelson MI, Turner PE, Patton JT. Reassortment in segmented RNA viruses: mechanisms and outcomes. Nat Rev Microbiol. 2016;14(7):448–60. 27211789 10.1038/nrmicro.2016.46 PMC5119462

[4] Desselberger U. Rotaviruses. Virus Res. 2014;190:75–96. 25016036 10.1016/j.virusres.2014.06.016

[5] Matthijnssens J, Otto PH, Ciarlet M, Desselberger U, Van Ranst M, Johne R. VP6-sequence-based cutoff values as a criterion for rotavirus species demarcation. Arch Virol. 2012;157(6):1177–82. 22430951 10.1007/s00705-012-1273-3

[6] Patton JT. Rotavirus diversity and evolution in the post-vaccine world. Discov Med. 2012;13(68):85–97. 22284787 PMC3738915

[7] Dhama K, Chauhan RS, Mahendran M, Malik SVS. Rotavirus diarrhea in bovines and other domestic animals. Vet Res Commun. 2009;33(1):1–23. 18622713 10.1007/s11259-008-9070-x PMC7088678

[8] Brunauer M, Roch FF, Conrady B. Prevalence of Worldwide Neonatal Calf Diarrhoea Caused by Bovine Rotavirus in Combination with Bovine Coronavirus, Escherichia coli K99 and Cryptosporidium spp.: A Meta-Analysis. Animals. 2021;11(4):1014. 10.3390/ani11041014 PMC8066230 33916839

[9] Singh S, Singh R, Singh KP, Singh V, Malik YPS, Kamdi B, et al. Immunohistochemical and molecular detection of natural cases of bovine rotavirus and coronavirus infection causing enteritis in dairy calves. Microb Pathog. 2020;138: 103814. 31639467 10.1016/j.micpath.2019.103814 PMC7127329

[10] Mawatari T, Taneichi A, Kawagoe T, Hosokawa M, Togashi K, Tsunemitsu H. Detection of a Bovine Group C Rotavirus from Adult Cows with Diarrhea and Reduced Milk Production. J Vet Med Sci. 2004;66(7):887–90. 15297766 10.1292/jvms.66.887

[11] Moya A, Holmes EC, González-Candelas F. The population genetics and evolutionary epidemiology of RNA viruses. Nat Rev Microbiol. 2004;2(4):279–88. 15031727 10.1038/nrmicro863 PMC7096949

[12] Woo PCY, Lau SKP, Huang Y, Yuen KY. Coronavirus Diversity, Phylogeny and Interspecies Jumping. Exp Biol Med. 2009;234(10):1117–27. 10.3181/0903-MR-94 19546349

[13] Zhang XW, Yap YL, Danchin A. Testing the hypothesis of a recombinant origin of the SARS-associated coronavirus. Arch Virol. 2005;150(1):1–20. 15480857 10.1007/s00705-004-0413-9 PMC7087341

[14] Smith EC, Denison MR. Implications of altered replication fidelity on the evolution and pathogenesis of coronaviruses. Curr Opin Virol. 2012;2(5):519–24. 22857992 10.1016/j.coviro.2012.07.005 PMC7102773

[15] Brandão PE, Gregori F, Sforsin AJ, Villarreal LYB, Jerez JA. Winter dysentery in cows associated with Bovine Coronavirus (BCoV). Arq Bras Med Veterinária E Zootec. 2007;59(4):1074–6.

[16] Stipp DT, Barry AF, Alfieri AF, Takiuchi E, Amude AM, Alfieri AA. Frequency of BCoV detection by a semi-nested PCR assay in faeces of calves from Brazilian cattle herds. Trop Anim Health Prod. 2009;41(7):1563–7. 19370396 10.1007/s11250-009-9347-2 PMC7089217

[17] Andersen KG, Rambaut A, Lipkin WI, Holmes EC, Garry RF. The proximal origin of SARS-CoV-2. Nat Med. 2020;26(4):450–2. 32284615 10.1038/s41591-020-0820-9 PMC7095063

[18] Gralinski LE, Menachery VD. Return of the Coronavirus: 2019-nCoV. Viruses. 2020;12(2):135. 31991541 10.3390/v12020135 PMC7077245

[19] Amer HM. Bovine-like coronaviruses in domestic and wild ruminants. Anim Health Res Rev. 2018;19(2):113–24. 30683171 10.1017/S1466252318000117 PMC7108644

[20] Korir L, Manning L, Moore HL, Lindahl JF, Gemechu G, Mihret A, et al. Adoption of dairy technologies in smallholder dairy farms in Ethiopia. Front Sustain Food Syst. 2023;12(7):1070349.

[21] Wong JT, Lane JK, Allan FK, Vidal G, Vance C, Donadeu M, et al. Reducing Calf Mortality in Ethiopia. Animals. 2022;12(16):2126. 36009716 10.3390/ani12162126 PMC9405078

[22] Ahmedin UM, Assen AA. Calf morbidity, mortality, and management practices in dairy farms in Jimma City, Southwestern Ethiopia. BMC Vet Res. 2023;19(1):249. 38017486 10.1186/s12917-023-03815-w PMC10683357

[23] Cho HC, Kim Y, Cho YI, Park J, Choi KS. Evaluation of bovine coronavirus in Korean native calves challenged through different inoculation routes. Vet Res. 2024;55(1):74. 38863015 10.1186/s13567-024-01331-9 PMC11165853

[24] Gomez DE, Arroyo LG, Poljak Z, Viel L, Weese JS. Detection of Bovine Coronavirus in Healthy and Diarrheic Dairy Calves. J Vet Intern Med. 2017;31(6):1884–91. 28913936 10.1111/jvim.14811 PMC5697193

[25] Izzo M, Kirkland P, Mohler V, Perkins N, Gunn A, House J. Prevalence of major enteric pathogens in Australian dairy calves with diarrhoea. Aust Vet J. 2011;89(5):167–73. 21495987 10.1111/j.1751-0813.2011.00692.x PMC7159393

[26] Pérez E, Kummeling A, Janssen MMH, Jiménez C, Alvarado R, Caballero M, et al. Infectious agents associated with diarrhoea of calves in the canton of Tilarán. Costa Rica Prev Vet Med. 1998;33(1–4):195–205. 9500174 10.1016/S0167-5877(97)00038-X PMC7134171

[27] Lanz Uhde F, Kaufmann T, Sager H, Albini S, Zanoni R, Schelling E, et al. Prevalence of four enteropathogens in the faeces of young diarrhoeic dairy calves in Switzerland. Vet Rec. 2008;163(12):362–6. 18806281 10.1136/vr.163.12.362

[28] Debelo M, Abdela H, Tesfaye A, Tiruneh A, Mekonnen G, Asefa Z, et al. Prevalence of Bovine Rotavirus and Coronavirus in Neonatal Calves in Dairy Farms of Addis Ababa, Ethiopia: Preliminary Study. Kumar B, editor. BioMed Res Int. 2021;2021:1–6. 10.1155/2021/5778455 PMC8595031 34796233

[29] Bergholm J, Tessema TS, Blomström AL, Berg M. Detection and molecular characterization of major enteric pathogens in calves in central Ethiopia. BMC Vet Res. 2024;20(1):389. 39227796 10.1186/s12917-024-04258-7 PMC11373192

[30] Seid U, Dawo F, Tesfaye A, Ahmednur M. Isolation and Characterization of Coronavirus and Rotavirus Associated with Calves in Central Part of Oromia, Ethiopia. Mancianti F, editor. Vet Med Int. 2020;2020:1–10. 10.1155/2020/8869970 PMC7723472 33335702

[31] Lang Y, Li W, Li Z, Koerhuis D, Van Den Burg ACS, Rozemuller E, et al. Coronavirus hemagglutinin-esterase and spike proteins coevolve for functional balance and optimal virion avidity. Proc Natl Acad Sci. 2020;117(41):25759–70. 32994342 10.1073/pnas.2006299117 PMC7568303

[32] Schultze B, Wahn K, Klenk HD, Herrler G. Isolated HE-protein from hemagglutinating encephalomyelitis virus and bovine coronavirus has receptor-destroying and receptor-binding activity. Virology. 1991;180(1):221–8. 1984649 10.1016/0042-6822(91)90026-8 PMC7131771

[33] Schultze B, Herrler G. Bovine coronavirus uses N-acetyl-9-O-acetylneuraminic acid as a receptor determinant to initiate the infection of cultured cells. J Gen Virol. 1992;73(4):901–6. 1321878 10.1099/0022-1317-73-4-901

[34] Schultze B, Herrler G. Recognition of cellular receptors by bovine coronavirus. In: Brinton MA, Calisher CH, Rueckert R, editors. Positive-Strand RNA Viruses [Internet]. Vienna: Springer Vienna; 1994 [cited 2024 Oct 10]. p. 451–9. Available from: http://link.springer.com/10.1007/978-3-7091-9326-6_44. 10.1007/978-3-7091-9326-6_44 8032275

[35] David D, Storm N, Ilan W, Sol A. Characterization of winter dysentery bovine coronavirus isolated from cattle in Israel. Viruses. 2021;13(6):1070. 34199933 10.3390/v13061070 PMC8226893

[36] Hasoksuz M, Sreevatsan S, Cho KO, Hoet AE, Saif LJ. Molecular analysis of the S1 subunit of the spike glycoprotein of respiratory and enteric bovine coronavirus isolates. Virus Res. 2002;84(1–2):101–9. 11900843 10.1016/S0168-1702(02)00004-7 PMC7127276

[37] Jeong JH, Kim GY, Yoon SS, Park SJ, Kim YJ, Sung CM, et al. Molecular analysis of S gene of spike glycoprotein of winter dysentery bovine coronavirus circulated in Korea during 2002–2003. Virus Res. 2005;108(1–2):207–12. 15681072 10.1016/j.virusres.2004.07.003 PMC7114273

[38] Zhang X, Hasoksuz M, Spiro D, Halpin R, Wang S, Vlasova A, et al. Quasispecies of bovine enteric and respiratory coronaviruses based on complete genome sequences and genetic changes after tissue culture adaptation. Virology. 2007;363(1):1–10. 17434558 10.1016/j.virol.2007.03.018 PMC7103286

[39] Zhang X, Kousoulas KG, Storz J. The hemagglutinin/esterase glycoprotein of bovine coronaviruses: Sequence and functional comparisons between virulent and avirulent strains. Virology. 1991;185(2):847–52. 1962455 10.1016/0042-6822(91)90557-R PMC7131179

[40] Abi K mo, Zhang Q, Zhang B, Zhou L, Yue H, Tang C. An emerging novel bovine coronavirus with a 4-amino-acid insertion in the receptor-binding domain of the hemagglutinin-esterase gene. Arch Virol. 2020 Dec;165(12):3011–5. 10.1007/s00705-020-04840-y PMC7538171 33025200

[41] Park JG, Kim HJ, Matthijnssens J, Alfajaro MM, Kim DS, Son KY, et al. Different virulence of porcine and porcine-like bovine rotavirus strains with genetically nearly identical genomes in piglets and calves. Vet Res. 2013;44(1):88. 24083947 10.1186/1297-9716-44-88 PMC3851489

[42] MacGillivray DM, Kollmann TR. The Role of Environmental Factors in Modulating Immune Responses in Early Life. Front Immunol. 2014;5. [cited 2024 Oct 11]. 10.3389/fimmu.2014.00434/abstract. 10.3389/fimmu.2014.00434 PMC4161944 25309535

[43] Saif LJ, Fernandez FM. Group A Rotavirus Veterinary Vaccines. J Infect Dis. 1996;174(Supplement 1):S98–106. 8752298 10.1093/infdis/174.Supplement_1.S98 PMC7110367

[44] Adah MI, Nagashima S, Wakuda M, Taniguchi K. Close Relationship between G8-Serotype Bovine and Human Rotaviruses Isolated in Nigeria. J Clin Microbiol. 2003;41(8):3945–50. 12904426 10.1128/JCM.41.8.3945-3950.2003 PMC179859

[45] Sircar S, Malik YS, Kumar P, Ansari MI, Bhat S, Shanmuganathan S, et al. Genomic Analysis of an Indian G8P[1] caprine rotavirus-a strain revealing artiodactyl and DS-1-like human multispecies reassortment. Front Vet Sci. 2021;27(7):606661. 10.3389/fvets.2020.606661 PMC7873603 33585597

[46] Heylen E, Zeller M, Ciarlet M, Lawrence J, Steele D, Van Ranst M, et al. Comparative analysis of pentavalent rotavirus vaccine strains and G8 rotaviruses identified during vaccine trial in Africa. Sci Rep. 2015;5(1):14658. 26440913 10.1038/srep14658 PMC4594120

[47] Matthijnssens J, Bilcke J, Ciarlet M, Martella V, Bányai K, Rahman M, et al. Rotavirus disease and vaccination: Impact on genotype diversity. Future Microbiol. 2009;4(10):1303–16. 19995190 10.2217/fmb.09.96

[48] Ghosh S, Kobayashi N, Nagashima S, Chawla-Sarkar M, Krishnan T, Ganesh B, et al. Molecular characterization of the VP1, VP2, VP4, VP6, NSP1 and NSP2 genes of bovine group B rotaviruses: identification of a novel VP4 genotype. Arch Virol. 2010;155(2):159–67. 19936611 10.1007/s00705-009-0555-x

[49] Ghosh S, Alam MM, Ahmed MU, Talukdar RI, Paul SK, Kobayashi N. Complete genome constellation of a caprine group A rotavirus strain reveals common evolution with ruminant and human rotavirus strains. J Gen Virol. 2010;91(9):2367–73. 20505013 10.1099/vir.0.022244-0

[50] Rahman M, Matthijnssens J, Saiada F, Hassan Z, Heylen E, Azim T, et al. Complete genomic analysis of a Bangladeshi G1P[8] rotavirus strain detected in 2003 reveals a close evolutionary relationship with contemporary human Wa-like strains. Infect Genet Evol. 2010;10(6):746–54. 20441801 10.1016/j.meegid.2010.04.011

[51] Steyer A, Sagadin M, Kolenc M, Poljšak-Prijatelj M. Whole genome sequence analysis of bovine G6P[11] rotavirus strain found in a child with gastroenteritis. Infect Genet Evol. 2013;13:89–95. 22995281 10.1016/j.meegid.2012.09.004

[52] Varghese V, Ghosh S, Das S, Bhattacharya SK, Krishnan T, Karmakar P, et al. Characterization of VP1, VP2 and VP3 Gene Segments of A Human Rotavirus Closely Related to Porcine Strains. Virus Genes. 2006;32(3):241–7. 16732476 10.1007/s11262-005-6908-y

[53] Park SJ, Jeong C, Yoon SS, Choy HE, Saif LJ, Park SH, et al. Detection and Characterization of Bovine Coronaviruses in Fecal Specimens of Adult Cattle with Diarrhea during the Warmer Seasons. J Clin Microbiol. 2006;44(9):3178–88. 16954245 10.1128/JCM.02667-05 PMC1594715

[54] Muyyarikkandy MS, Kniel K, Bower WA, Vieira AR, Negrón ME, Thakur S. Assessment of critical gaps in prevention, control, and response to major bacterial, viral, and protozoal infectious diseases at the human, animal, and environmental interface. In: Modernizing Global Health Security to Prevent, Detect, and Respond [Internet]. Elsevier; 2024 [cited 2024 Oct 11]. p. 175–95. Available from: https://linkinghub.elsevier.com/retrieve/pii/B9780323909457000245.

[55] Bwogi J, Karamagi C, Byarugaba DK, Tushabe P, Kiguli S, Namuwulya P, et al. Co-Surveillance of rotaviruses in humans and domestic animals in central Uganda reveals circulation of wide genotype diversity in the animals. Viruses. 2023;15(3):738. 36992447 10.3390/v15030738 PMC10052166

[56] Cholleti H, De Jong J, Blomström AL, Berg M. Characterization of pipistrellus pygmaeus bat virome from Sweden. Viruses. 2022;14(8):1654. 36016275 10.3390/v14081654 PMC9415950

[57] Decaro N, Elia G, Campolo M, Desario C, Mari V, Radogna A, et al. Detection of bovine coronavirus using a TaqMan-based real-time RT-PCR assay. J Virol Methods. 2008;151(2):167–71. 18579223 10.1016/j.jviromet.2008.05.016 PMC7112840

[58] Fukuda M, Kuga K, Miyazaki A, Suzuki T, Tasei K, Aita T, et al. Development and application of one-step multiplex reverse transcription PCR for simultaneous detection of five diarrheal viruses in adult cattle. Arch Virol. 2012;157(6):1063–9. 22407445 10.1007/s00705-012-1271-5 PMC7086690

[59] Gómara MI, Cubitt D, Desselberger U, Gray J. Amino acid substitution within the VP7 Protein of G2 rotavirus strains associated with failure to serotype. J Clin Microbiol. 2001;39(10):3796–8. 11574622 10.1128/JCM.39.10.3796-3798.2001 PMC88438

[60] Mijatovic-Rustempasic S, Bányai K, Esona MD, Foytich K, Bowen MD, Gentsch JR. Genome sequence based molecular epidemiology of unusual US Rotavirus A G9 strains isolated from Omaha, USA between 1997 and 2000. Infect Genet Evol. 2011;11(2):522–7. 21130184 10.1016/j.meegid.2010.11.012

[61] Matthijnssens J, Rahman M, Martella V, Xuelei Y, De Vos S, De Leener K, et al. Full genomic analysis of human rotavirus strain B4106 and lapine rotavirus strain 30/96 provides evidence for interspecies transmission. J Virol. 2006;80(8):3801–10. 16571797 10.1128/JVI.80.8.3801-3810.2006 PMC1440464

[62] Brandão PE, Gregori F, Richtzenhain LJ, Rosales CAR, Villarreal LYB, Jerez JA. Molecular analysis of Brazilian strains of bovine coronavirus (BCoV) reveals a deletion within the hypervariable region of the S1 subunit of the spike glycoprotein also found in human coronavirus OC43. Arch Virol. 2006;151(9):1735–48. 16583154 10.1007/s00705-006-0752-9 PMC7086848

[63] Pickett BE, Sadat EL, Zhang Y, Noronha JM, Squires RB, Hunt V, et al. ViPR: an open bioinformatics database and analysis resource for virology research. Nucleic Acids Res. 2012;40(D1):D593–8. 22006842 10.1093/nar/gkr859 PMC3245011

[64] Kumar S, Stecher G, Li M, Knyaz C, Tamura K. MEGA X: Molecular Evolutionary Genetics Analysis across Computing Platforms. Battistuzzi FU, editor. Mol Biol Evol. 2018;35(6):1547–9. 10.1093/molbev/msy096 PMC5967553 29722887

