# Supplementary material for: Detection and characterization of bovine coronavirus and rotavirus in calves in Ethiopia
Source: BMC Vet Res. 2025 Feb 28;21:122. doi: 10.1186/s12917-025-04563-9 (PMC11869714; doi:10.1186/s12917-025-04563-9)
Supplement: Supplementary file 1 — Supplementary Material 1. [file 12917_2025_4563_MOESM1_ESM.docx]

**Supplementary material .1 Sequenceses used for phylogenetic analysis**

**G8 rotavirus**

**Accession number Host Country Collection date genotype**

MT501457 Caprine IND 2015 G8P1

MT501457.1 Caprine IND 2015 G8P1

JX156636.1 human TWN 2008 G8P14

MK519593.1 Caprine BGD G8P1

GU984760.1 Bovine IND 2007 G8P14

PQ246061 Bovine ETH 2023 G8

LC133519.1 Bovine THA 1988 G8P1

GU984761.1 Bovine IND 007 G8P14

LC119109.1 Bovine NGA 1998 G8P1

AF361439 Bovine NGR G8

KP882714.1 Human Kenya 2008 G8P6

FJ386444.1 Simian KEN 1999 G8P6

GQ225781 Bovine USA 2003 G8

AB749167.1 Human MWI 2002 G8P4

KX212865.1 Bovine TUN 2015 G8P5

KC257096.1 Camel SDN 2002 G8P11

U14999.1 Bovine USA 1969 G8P1

LC656030 Bovine JPN 2019 G8P1

L20883.1 Bovine GBR 1986 G8P5

JN831225.1 Bovine ZAF 2007 G8P1

MH016182.1 Bovine BRA 2017 G8P11

DQ838598.1 Bovine ZAF 1965 G8P1

JF693045 bovine ZAF 1965 G8P1

EF554153.1 Sheep ESP 2002 G8P14

AB077054 Bovine JPN 2002 G8

FJ206051.1 Bovine KOR 2006 G8P7

FJ206055.1 Bovine KOR 2006 G8P7

AB158431. Bovine JPN 2000 G8P14

FJ347105.1 Guanaco ARG 1999 G8P14

KF577843.1 Caprine ARG 2011 G8P1

LC553633.1 Bovine JPN 2007 G10P11

LC133552.1 Bovine USA 1983 G10P11

**G10 rotavirus**

**Accession number Host Country Collection date genotype**

KP882714.1 Human Keny 2008 G8P6

OR253974 Bovine ARG 2008 G10P11

KC895810 Bovine ARG 1999 G10P5

MN194497 Human KEN 2011 G10P8

KJ753644 Human KEN G1G10P8

HQ315854 Bovine HUN 2002 G10

KU729665 Bovine MOR 2014 G10

KT461288 Goat MOR 2014 G10

EU548032 giraffe IRL G10

MG269496B Bovine BRA 2013 G10PX

HQ419066 camel Egypt 2004 G10

LC319667 Human NGR 2014

LC319666 Human NGR 2014 G10P10

MK638904 Yak CHN 2017 G10PX

OP793923 KOR 2016 G10P11

OQ735384 Bovine Vietnam 2018 G10P11

OQ735383 Bovine Vietnam 2018 G10P11

MG269495 Bovine BRA 2014 G10P11

MG269486 Bovine BRZ 2013 G10P11

KC215545 Vaccine USA 2009 G10P5

LC553633 Bovine JPN 2007 G10P11

KP013395 Bovine IRAN G10

LC133552.1 Bovine USA 1983 G10P11

MN928499 Bovine CHN 2018 G10PX

GQ433985 Bovine IRL 2009

OP377078 Bovine Egypt 2019

KX268316 Bovine Egypt 2015 G10

PP417702.1 Bovine ETH 2023 G10

PQ246063 Bovine ETB 2023

PQ246062 Bovine ETH 2023 G10

KX599295 Bovine TUN 2014 G10P11

KX599294 Bovine TUN 2014 G10P11

MN583317 Goat MOR 2012 G10P14

KU729666 Bovine MOR 2014 G10

KP013390 Bovine IRN G10

KM609894 Bovine IRN 2011 G10/

LC594507 Bovine JPN 2019 G10P11

LC656034 Bovine JPN 2020 G10P11

LC591077 Bovine Japan 2017 G10P6

**BCoV_HE gene**

**Accession number Host Country Collection date**

OR271248.1 Bovine Ireland 2023

PP156989.1 Bovine Ireland 2024

PP534473.1 Marmot Italy 2024

PP156979.1 Bovine Albania 2023

PP156981.1 Bovine Albania 2023

PP156982.1 Bovine Albania 2023

MW310543.1 Bovine Israel 2019

OQ507475.1 Bovine Norway 2023

MW310545.1 Bovine Israel 2021

MW310542.1 Bovine Israel 2021

KX982264.1 Bovine France 2014

EU019216.1 Bubalus Italy

EF445634.1 Bovine Italy 2007

ON792943.1 Bovine Ireland 2022

ON014593.1 Dama_dama UK 2023

MG757142.1 Bovine France 2019

MG757141.1 Bovine France

MG757139.1 Bovine France 2019

MG757139.1 Bovine France 2019

MW310546.1 Bovine Israel 2017

PQ268636 Bovine Ethiopia 2023

PQ268636 Bovine Ethiopia 2023

PQ268636 Bovine Ethiopia 2023

MW711315.1 Bovine China 2021

MW711311.1 Bovine China 2021

MW711310.1 Bovine China 2021

MH203060.1 Bovine VietNam 2018

MH203061.1 Bovine VietNam 2018

MT543033.1 Canada 2021

MK045992.1 Bovine VietNam 2019

OP037439.1 Bovine USA 2022

MZ100070.1 Tapir Canada 2021

OR502442.1 Bovine USA 2024

MT543034.1 Bovine Canada 2021

LC494160.1 Bovine Japan 2020

LC494174.1 Bovine Japan 2020

LC494136.1 Bovine Japan 2020

EF424620.1 Bovine USA 2007

OP186340.1 Bovine South_Korea 2022

OP186330.1 Bovine South_Korea 2022

AH014875.2 Bovine 2005

DQ994167.1 Bovine SouthKorea 2006:

KU558922.1 Bubalus bubalis Bangladesh 2016

KU558923.1 Buffalo Bangladesh 2016

KF906251.1 camel UAE 014

KT368891.1 Camel Saudi_Arabia 2015

KF906250.1 Camel UAE 2014

MF593476.1 Camel UAE 2017

FJ415324.1 Human

DQ682406.1 Canine UK 2007

MH249786.1 Canine New_Zealand 2019

OP820540.1 Bovine IND 2022

U00735.2/

OP296992.1 Bovine Taiwan 2022

AF220295.1 Bovine Quebec 2001

OP820530. Bubalus bubalis IND 2021

LQ289124.1/1

**BcoV S1 HVR**

**Accession number Host Country Collection date**

| PP156983.1 | Bos taurus |  | Ireland | 2022 |
| --- | --- | --- | --- | --- |
| PP156978.1 | Bos taurus |  | Albania | 2023 |
| PP156979.1 | Bos taurus |  | Albania | 2023 |
| KP059127.1 | Bovine |  | Argentina | 2011 |
| KY612617.1 | Cattle |  | Austria |  |
| KY612618.1 | Cattle |  | Austria |  |
| KU558923.1 | Buffalo |  | Bangladesh | 2014 |
| KT381476.1 | Bovine |  | Brazil | 2014 |
| OM632714.1 | Bovine |  | Brazil | 2018 |
| MZ100070.1 | Tapir |  | Canada | 2021 |
| AH010363.2 | Bovine |  | Canada |  |
| OR947444.1 | Cattle |  | China | 2020 |
| MW521190.1 | Bovine |  | China | 2020 |
| KM677163.1 | Cattle |  | Croatia | 2011 |
| KM677164.1 | Cattle |  | Croatia | 2011 |
| KF169914.1 | Calf |  | Denmark | 2003 |
| KF169919.1 | Cattle |  | Denmark | 2005 |
| KM386671.1 | Cattle |  | Egypt |  |
| MN531695.1 | Cattle |  | Egypt | 2019 |
| MN531697.1 | Cattle |  | Egypt | 2019 |
| KT318117.1 | Bovine |  | France | 2012 |
| KT318118.1 | Bovine |  | France | 2013 |
| KT318119.1 | Bovine |  | France | 2013 |
| MG757138.1 | Cattle |  | France | 2014 |
| MG757144.1 | Cattle |  | France | 2014 |
| FJ415324.1 | Human |  | Germany | 1988 |
| OP820541.1 | Bos indicus |  | India | 2022 |
| MH371011.1 | bovine calf |  | Iran | 2009 |
| MK932864.1 | Calf |  | Iran | 2018 |
| MK932865.1 | Calf |  | Iran | 2019 |
| ON792956.1 | Charolais |  | Ireland | 2019 |
| OR271252.1 | Bos taurus |  | Ireland | 2022 |
| MW310532.1 | Bovine |  | Israel | 2018 |
| MW310535.1 | Bovine |  | Israel | 2020 |
| MW310538.1 | Bovine |  | Israel | 2019 |
| MW310539.1 | Bovine |  | Israel | 2019 |
| EU019216.1 | Bubalus bubalis |  | Italy |  |
| EU814647.1 | Cattle |  | Italy |  |
| AB354579.1 |  |  | Japan |  |
| LC494138.1 |  |  | Japan | 2015 |
| OQ507475.1 | Cattle |  | Norway | 2012 |
| KX266948.1 | Vicugna pacos |  | Peru | 2014 |
| KX266949.1 | Vicugna pacos |  | Peru | 2014 |
| KY612619.1 | Cattle |  | Slovakia |  |
| KY612620.1 | Cattle |  | Slovakia |  |
| OP186326.1 | Bovine |  | South korea | 2017 |
| EU401988.1 |  |  | SouthKorea |  |
| KF169908.1 | Cattle |  | Sweden | 1992 |
| KF169911.1 | Calf |  | Sweden | 2002 |
| KX373886.1 | Bos taurus |  | Thailand | 2015 |
| KX373888.1 | Bos taurus |  | Thailand | 2015 |
| MK989626.1 | cattle |  | Turkey | 2015 |
| MK989633.1 | cattle |  | Turkey | 2016 |
| ON014593.1 | Dama dama |  | UK | 2019 |
| MK318157.1 | cattle |  | Uruguay | 2016 |
| MK318174.1 | cattle |  | Uruguay | 2016 |
| DQ320764.1 |  |  | USA |  |
| FJ425186.1 | waterbuck |  | USA | 1994 |
| MH203065.1 | Calf |  | Viet_Nam | 2017 |
| MK046011.1 | bovine |  | Viet_Nam | 2018 |
| U00735.2 |  |  |  |  |
| Z32769.1 |  |  |  |  |
